# Supplementary material for: Global profiling of distinct cysteine redox forms reveals wide-ranging redox regulation in C. elegans
Source: Nat Commun. 2021 Mar 3;12:1415. doi: 10.1038/s41467-021-21686-3 (PMC7930113; doi:10.1038/s41467-021-21686-3)
Supplement: Supplementary file 1 — Supplementary Information [file 41467_2021_21686_MOESM1_ESM.pdf]

SUPPLEMENTARY INFORMATION

Global profiling of distinct cysteine redox forms reveals wide-ranging redox regulation in *C. elegans*

Jin Meng[1,2,3,8], Ling Fu[4,5,8], Keke Liu[4], Caiping Tian[4,6], Ziyun Wu[1,2,3], Youngeun Jung[7], Renan B. Ferreira[7], Kate S. Carroll[7], T. Keith Blackwell[1,2,3*], Jing Yang[4,5*]

1. Research Division, Joslin Diabetes Center, Boston, MA 02215, USA

2. Department of Genetics, Harvard Medical School, Boston, MA 02115 USA

3. Harvard Stem Cell Institute, Cambridge, MA 02138, USA

4. State Key Laboratory of Proteomics, Beijing Proteome Research Center, National Center for Protein Sciences • Beijing, Beijing Institute of Lifeomics, Beijing 102206, China

5. Innovation Institute of Medical School, Medical College, Qingdao University, Qingdao 266071, China

6. School of Medicine, Tsinghua University, Beijing 100084, China

7. Department of Chemistry, The Scripps Research Institute, Jupiter, Florida 33458, USA

8. These authors contributed equally to this work

[*] Correspondence: keith.blackwell@joslin.harvard.edu or yangjing54@hotmail.com

a

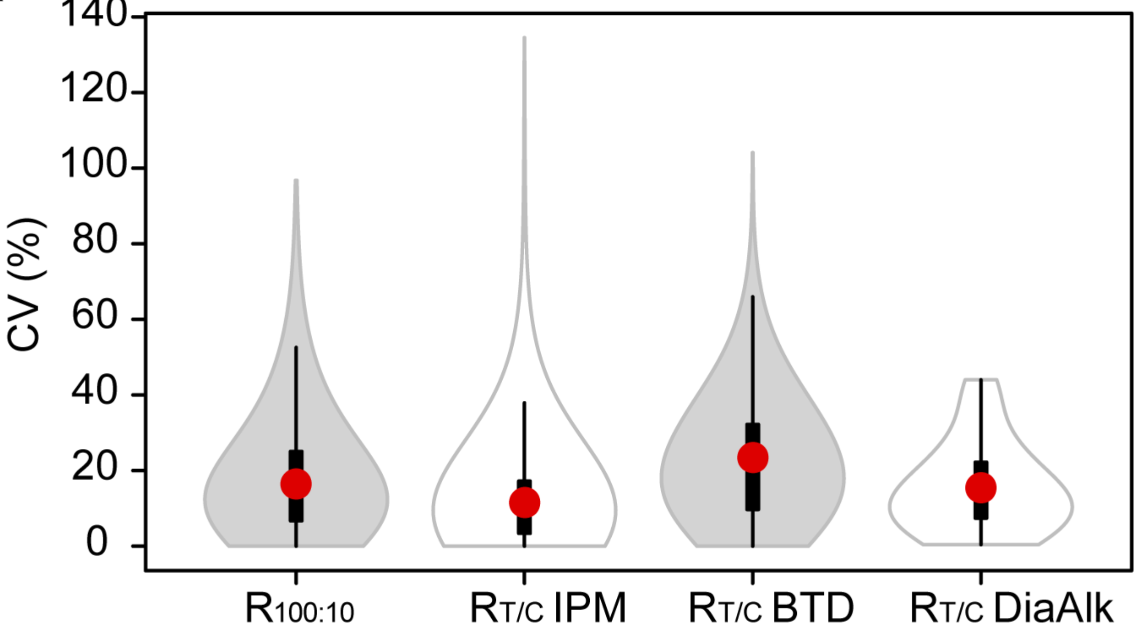

b

Statistics	$R_{100:10}$	$R_{T/C}$ IPM	$R_{T/C}$ BTD	$R_{T/C}$ DiaAlk
Upper whisker	52.53	37.9	65.4	44
3rd quartile	25.05	17.2	32.2	22.2
Median	13.41	8.4	20.4	12.4
1st quartile	6.71	3.4	9.7	7.4
Lower whisker	0	0	0	0.4
Nr. of data points	3029	2623	657	21

Supplementary Fig. 1. Reproducibility of quantitative chemoproteomics. (**a**) Violin plots of CV values of all the ratio values for quantifiable cysteines from at least two biological replicates. (**b**) Statistics defining Violin plots shown in (**a**).

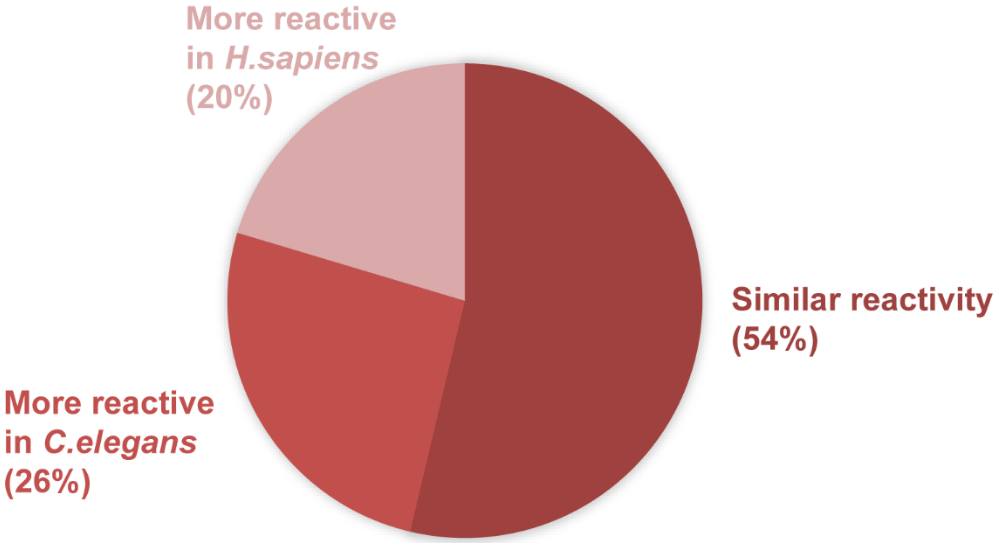

Supplementary Fig. 2. Pie chart showing the classification of conserved cysteines of which intrinsic reactivity was detected in both *C.elegans* and *H.sapiens*. Those conserved cysteines with similar reactivity in both species of are shown in dark red (with a fold-change between $R_{100:10}^{C.elegans}$ and $R_{100:10}^{H.sapiens}$ less than 1.5). Conserved cysteines exhibiting higher reactivity in *C.elegans* are shown in red, and those exhibiting higher reactivity in *H.sapiens* in light red. An intrinsic cysteine reactivity dataset of *H.sapiens* was retrieved from a previous study[1].

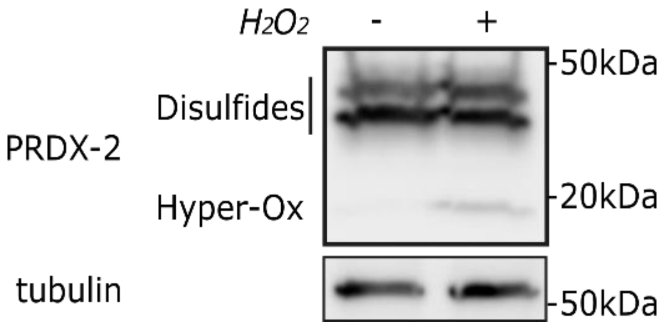

Supplementary Fig. 3. Immunoblots of PRDX-2 and tubulin with or without 1 mM H_2O_2 treatment for 5 min, showing that the reactive cysteines in the peroxiredoxin PRDX-2 were predominantly disulfide-linked under both conditions, which is consistent with previous reports[2]. n=2 experiments. Lysates were loaded twice onto gels for detection with different antibodies, and blots were processed in parallel.

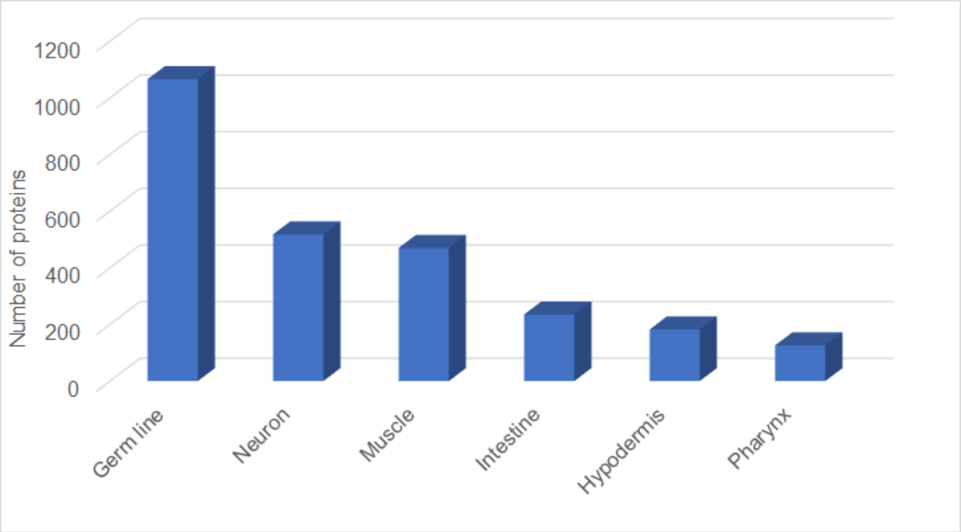

Supplementary Fig. 4. Tissue distribution of *C. elegans* proteins bearing oxidation-sensitive cysteines.

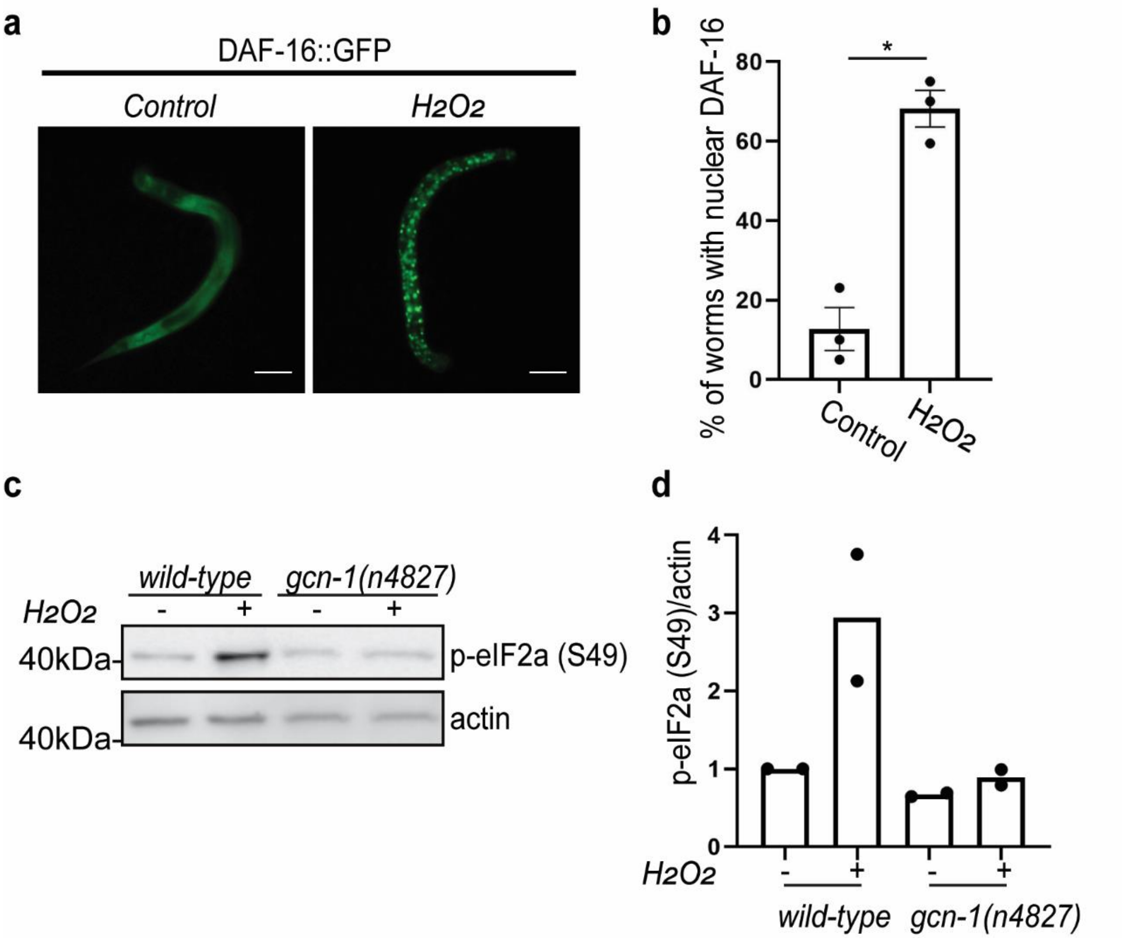

Supplementary Fig. 5. H_2O_2 treatment induces DAF-16::GFP nuclear accumulation and GCN-1-dependent eIF2α phosphorylation. (**a**) Fluorescent images and quantification (**b**) showing nuclear localization of DAF-16::GFP with or without 1 mM H_2O_2 (mean +/- SEM, n=3 experiments, at least 117 worms per condition). *, P=0.0308; Two-tailed Student's t-test. Scale bar=100 μm. (**c**) Representative Western blots and quantification (**d**) showing eIF2a phosphorylation levels in response to 1 mM H_2O_2 treatment in wild-type and *gcn-1(-)* animals (mean, n=2 experiments). Lysates were loaded onto two different gels for detection with different antibodies, and blots were processed in parallel.

a

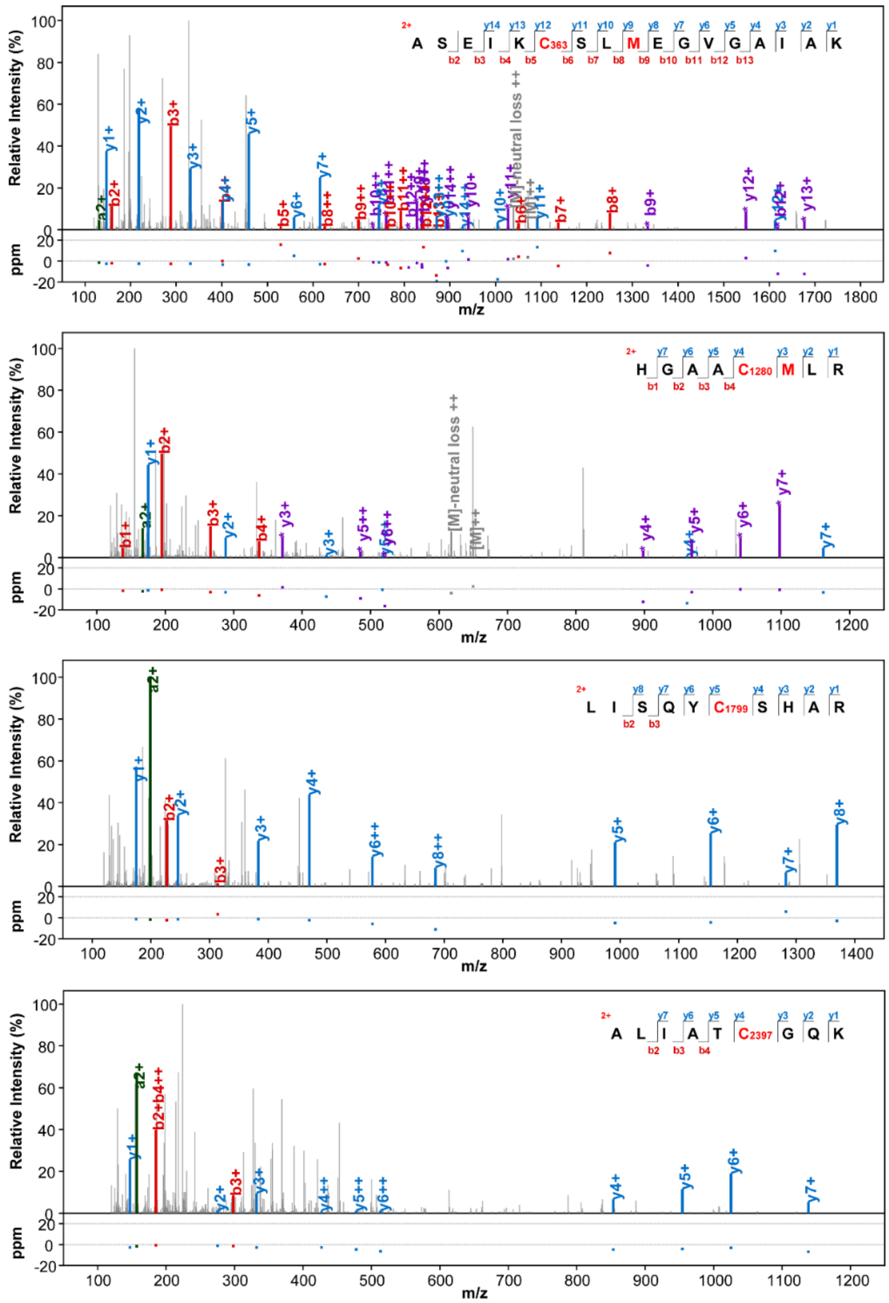

A S E I K C363 S L M E G V G A I A K
H G A A C1280 M L R
L I S Q Y C1799 S H A R
A L I A T C2397 G Q K
Relative Intensity (%)
ppm
m/z

b

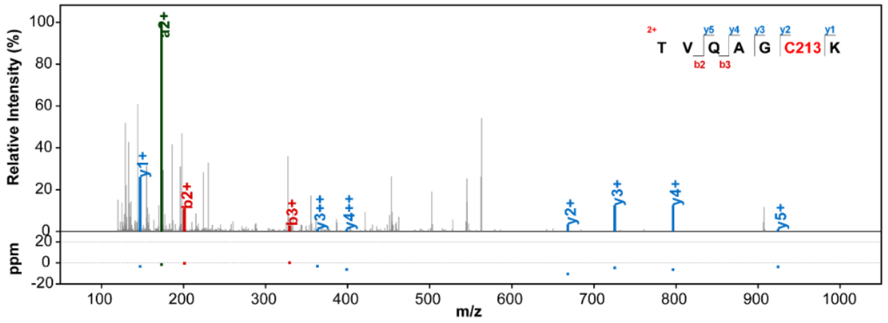

T V Q A G C213 K
Relative Intensity (%)
ppm
m/z

Supplementary Fig. 6. Cysteines that were identified as sulfenylated in GCN-1 (a) and SEK-1 (b). Sequence-specific b- and y-type ions are annotated in dark red and dark blue (methionine-containing y-type ions with a neural loss of 64 Da are shown in purple), enabling precise localization of the BTD-derived modification sites within each peptide sequence.

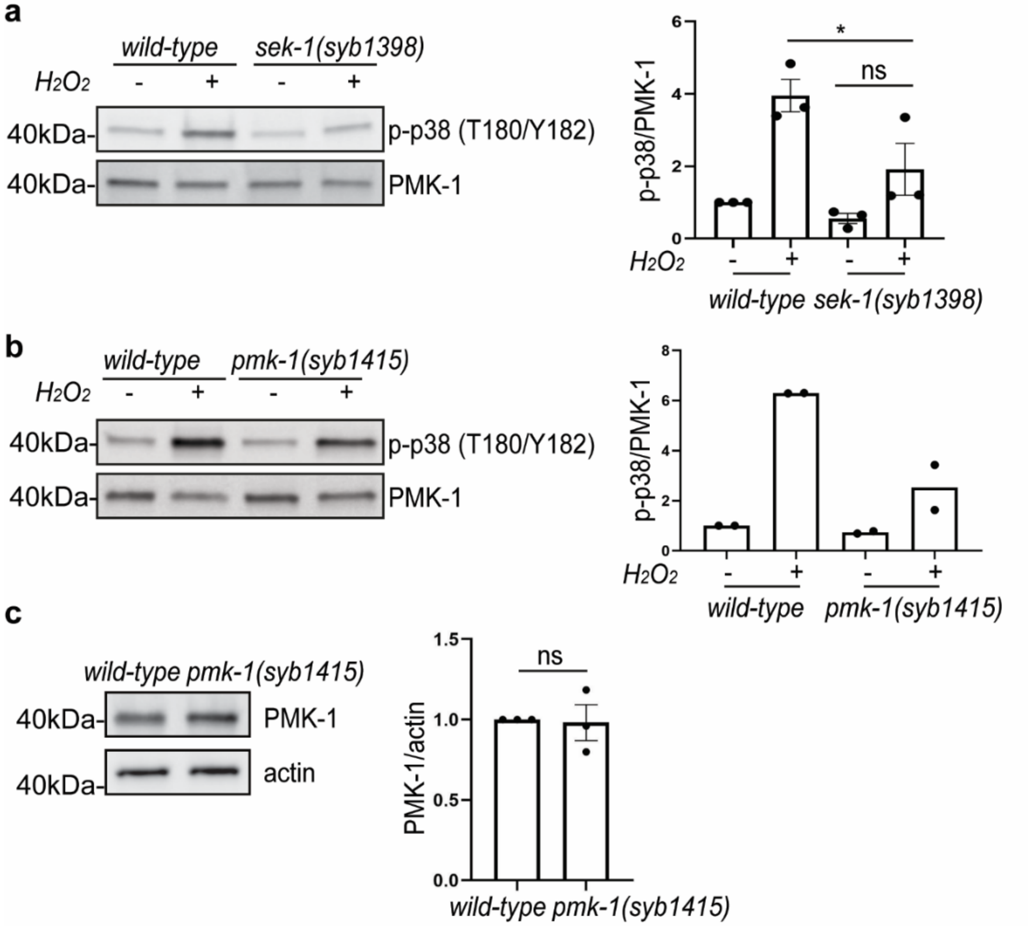

Supplementary Fig. 7. Cysteines in SEK-1 and PMK-1 are important for p38 signaling activity. (**a**) Representative Western blots and quantification showing levels of phosphorylated and total PMK-1/p38 in wild-type and SEK-1 C213S *(syb1398)* mutants with or without H_2O_2 (mean +/- SEM, n=3 experiments). One-way ANOVA with Bonferroni post-test. *, P=0.0199; ns, not significant (P=0.1110). (**b**) Immunoblots and quantification showing levels of phosphorylated and total PMK-1/p38 in wild-type and PMK-1 C173S *(syb1415)* mutant animals with or without H_2O_2 (mean, n=2 experiments). (**c**) Representative Western blots and quantification showing PMK-1/p38 protein levels in wild-type and the PMK-1 C173S mutant strain *(syb1415)* (mean +/- SEM, n=3 experiments). Two-tailed Student's t-test. ns, not significant (P=0.8834). Lysates were loaded onto two different gels for detection with different antibodies, and blots were processed in parallel.

Supplementary Table 1. Summary of the survival data in Figure 5g.

Genotype	Number of worms assayed	Median survival time (hours)	Maximal survival time (hours)
Wild-type (N2)	368	144	192
Pmk-1(syb1415)	314	144	192
Sek-1(syb1398)	369	120	144
Pmk-1(km25)	280	72	96
Sek-1(km4)	388	48	72

Supplementary Table 2: List of oligonucleotides used in this study

pmk-1 PCR primer Forward	TGATGGGAGCTGATCTATCAAATAT
pmk-1 PCR primer Reverse	TGAATTATCTTAGTCCATATTTTGG
sek-1 PCR primer Forward	GGCCAAGGTACGGAAAATCGATAAA
sek-1 PCR primer Reverse	TTTGCGTAGGGATGGGTGCCAACGG
pmk-1 sgRNA1	GAATTGAAAATTCTTGATTTTGG
pmk-1 sgRNA2	TTAGTCTATCAAATTCTTCGTGG
pmk-1 repair template:	TACTTCAAATCTTACAAACATGATTCCAGGTAACG GAGCCAATGTTTCCACAGACAACAATGGATCATAT ACTTCATCCGACTCCACGAGAAGGATACTATGTC GTTGAGCTTAATCGAAGTGTCTGGGTTGTTCCAAA TTATTATATTAACTTGACTCCAATTGGTACCGGAG CATATGGAACTGTTTGGTATGTCATTTCAACTGAT TTTTACTTTATTTTTCGATTTTTAGTGCTGCTGAAT GTACTCGCTCGGGGACTCGCGTCGCAATCAAAAA ATTCAATCGTCCATTTCAATCAATAATTCATGCCC GTCGTACATATCGTGAACTCCGACTTCTTCGTTGT ATGTGTCATGAAAATATAATTGATCTACTTGATGTC TTCACTCCAAATGAGAATGTAAATGATATCGAGGA TGTGTATTTTGTATCAATGTTGATGGGAGCTGATC TATCAAATATTCTCAAAATTCAGCGACTCAACGAT GACCACATTCAATTCTTAGTCTATCAAATTCTCCG TGGACTCAAATACATTCATTCTGCAGATATTATTCA TCGAGATCTTAAACCATCAAATATTGCGGTTAACG AGGATTCCGAATTGAAAATTCTAGATTTTGGATTG GCACGTCAAACTGATTCTGAAATGACTGGATACGT GGCAACAAGATGGTACAGAGCTCCAGAGATTATG

	CTCAATTGGATGCATTACACGCAGACTGTTGATGT ATGGTCAGTTGGGTGTATTCTTGCAGAACTTATCA CTGGAAAAACATTGTTCCCTGGATCTGATCGTAAG ACTTTTTTTCTACAATATTCCAAATATCTTCAATTTC CAAGCAAAAAACCAGAGTAAATATTTATGGTTGTT AACTTTATTGTACACTGGCGTTTCATAACATAGAA AAAAAATCATTTTGCTAAGTTATTCGTACCGTAGC AGGCGGAAAAAATTTATAATTAATTATTACCTCTTA TTGCAAATTTTGGTAGTTTATCTCCAGTTGATTTGA GGATTTAGGCGCATTTGTAGTCGCGGTGTTAACT ACCAAAATATGGACTAAGATAATTCAATAAAAACA AACAAAACAACAAAAAACAAAACACAAACGTTATT TTCTTTTGAAAATGTAGACTTTTCATTTAAAACTAC TGTTTCAGACATCGATC
sek-1 sgRNA	CCTTACATGCCACCAGAGAGAAT
sek-1 repair template	AATTTCAAAATATATATAGTGTTTCGATTTACAGAC TGTACAAGCCGGATCCAAACCTTACATGCCACCT GAGAGAATTGATGGAGAGACTAAGTCAGCCTATG ATGTGAGAGCTGATGT
act-1 qRT-PCR Forward	GCTGGACGTGATCTTACTGATTACC
act-1 qRT-PCR Reverse	GTAGCAGAGCTTCTCCTTGATGTC
gst-10 qRT-PCR Forward	GTCTACCACGTTTTGGATGC
gst-10 qRT-PCR Reverse	ACTTTGTCGGCCTTTCTCTT

References

1 Weerapana, E. *et al.* Quantitative reactivity profiling predicts functional cysteines in proteomes. *Nature* **468**, 790-795, doi:10.1038/nature09472 (2010).

2 Hourihan, J. M., Moronetti Mazzeo, L. E., Fernandez-Cardenas, L. P. & Blackwell, T. K. Cysteine Sulfenylation Directs IRE-1 to Activate the SKN-1/Nrf2 Antioxidant Response. *Mol Cell* **63**, 553-566, doi:10.1016/j.molcel.2016.07.019 (2016).
