# Supplementary material for: Global profiling of distinct cysteine redox forms reveals wide-ranging redox regulation in C. elegans
Source: Nat Commun. 2021 Mar 3;12:1415. doi: 10.1038/s41467-021-21686-3 (PMC7930113; doi:10.1038/s41467-021-21686-3)
Supplement: Supplementary file 3 — Description of Additional Supplementary Files [file 41467_2021_21686_MOESM3_ESM.pdf]

Description of Additional Supplementary Files

File Name: Supplementary Data 1

Description: Quantitative chemoproteomic analysis of intrinsic reactivity of the *C. elegans* cysteinome. Protein lysates from *C. elegans* were labeled with 10 and 100 µM IPM, respectively, and subjected to tryptic digestion. The resulting IPM-modified peptides were further conjugated to light and heavy azido biotin reagents with a photocleavable linker (Az-UV-biotin), respectively, via copper catalyzed alkyne-azide cycloaddition reaction (CuAAC, also known as click chemistry). The light and heavy labeled samples then were mixed equally in amount, cleaned with SCX and subjected to streptavidin-based enrichment. After several washing steps, the modified peptides were selectively eluted from beads under 365 nm UV light and subjected to LC-MS/MS-based proteomic analysis. Hyperreactive cysteines would be expected to label to completion at low probe concentrations (10 µM) and less reactive cysteines should show concentration-dependent increases in IPM labeling.

File Name: Supplementary Data 2

Description: Proteome-wide mapping of oxidation-sensitive cysteines in *C. elegans*. Protein lysates obtained from *C. elegans* were treated with or without H_2O_2 (5 mM, 5 min) and labeled with 100 µM of IPM. The IPM-labeled proteome was then processed into tryptic peptides. The resulting probe-labeled peptides were conjugated with both light and heavy azido-UV-cleavable-biotin (Az-UV-biotin) reagents (1:1) via CuAAC. The light and heavy 'Click' reaction mixtures were cleaned with SCX. The biotinylated peptides were captured with streptavidin and photoreleased for LC-MS/MS-based identification and quantification. High $R_{H/L}$ values are indicative of thiols that are less available after H_2O_2 treatment, suggesting potential redox-sensitive targets.

File Name: Supplementary Data 3

Description: Quantitative S-sulfenylome analysis of *C. elegans*. Protein lysates obtained from *C. elegans* were treated with or without H_2O_2 (5 mM, 5 min) and labeled with 5 mM BTD. The BTD-labeled proteome was then processed into tryptic peptides. The resulting probe-labeled peptides were conjugated with both light and heavy azido-UV-cleavable-biotin (Az-UV-biotin) reagents (1:1) via CuAAC. The light and heavy 'Click' reaction mixtures were cleaned with SCX. The biotinylated peptides were captured with streptavidin and photoreleased for LC-MS/MS-based identification and quantification.

File Name: Supplementary Data 4

Description: Quantitative S-sulfinylome analysis of *C. elegans*. Protein lysates obtained from *C. elegans* were treated with or without H_2O_2 (5 mM, 5 min) and labeled with 5 mM DiaAlk. The DiaAlk -labeled proteome was then processed into tryptic peptides. The resulting probe-labeled peptides were conjugated with both light and heavy azido-UV-cleavable-biotin (Az-UV-biotin) reagents (1:1) via CuAAC. The light and heavy 'Click' reaction mixtures were cleaned with SCX. The biotinylated peptides were captured with streptavidin and photoreleased for LC-MS/MS-based identification and quantification.

File Name: Supplementary Data 5

Description: The peroxide-sensitive *C. elegans* redoxome: 1537 proteins that changed dramatically in redox status upon peroxide treatment ($R_{T/C}^{IPM} \leq 0.67$, $R_{T/C}^{BTD} \geq 1.5$, or $R_{T/C}^{DiaAlk} \geq 1.5$).

File Name: Supplementary Data 6

Description: Cysteine residues in selective chromatin-modifying enzymes, RNA-binding proteins, translation factors, and cytosolic ribosomal proteins that exhibited dramatic changes in redox status ($R_{T/C}^{IPM} \leq 0.67$, $R_{T/C}^{BTD} \geq 1.5$, or $R_{T/C}^{DiaAlk} \geq 1.5$) after H_2O_2 treatment.
