# Supplementary material for: Global profiling of distinct cysteine redox forms reveals wide-ranging redox regulation in C. elegans
Source: Nat Commun. 2021 Mar 3;12:1415. doi: 10.1038/s41467-021-21686-3 (PMC7930113; doi:10.1038/s41467-021-21686-3)
Supplement: Supplementary file 10 — Reporting Summary [file 41467_2021_21686_MOESM10_ESM.pdf]

nature research

Corresponding author(s): Jing Yang, T.Keith Blackwell

Last updated by author(s): Jan 31, 2021

Reporting Summary

Nature Research wishes to improve the reproducibility of the work that we publish. This form provides structure for consistency and transparency in reporting. For further information on Nature Research policies, see our Editorial Policies and the Editorial Policy Checklist.

Statistics

For all statistical analyses, confirm that the following items are present in the figure legend, table legend, main text, or Methods section.

n/a	Confirmed	
☐	☒	The exact sample size (*n*) for each experimental group/condition, given as a discrete number and unit of measurement
☐	☒	A statement on whether measurements were taken from distinct samples or whether the same sample was measured repeatedly
☐	☒	The statistical test(s) used AND whether they are one- or two-sided<br>*Only common tests should be described solely by name; describe more complex techniques in the Methods section.*
☒	☐	A description of all covariates tested
☐	☒	A description of any assumptions or corrections, such as tests of normality and adjustment for multiple comparisons
☐	☒	A full description of the statistical parameters including central tendency (e.g. means) or other basic estimates (e.g. regression coefficient) AND variation (e.g. standard deviation) or associated estimates of uncertainty (e.g. confidence intervals)
☐	☒	For null hypothesis testing, the test statistic (e.g. F, t, r) with confidence intervals, effect sizes, degrees of freedom and P value noted<br>*Give P values as exact values whenever suitable.*
☒	☐	For Bayesian analysis, information on the choice of priors and Markov chain Monte Carlo settings
☒	☐	For hierarchical and complex designs, identification of the appropriate level for tests and full reporting of outcomes
☒	☐	Estimates of effect sizes (e.g. Cohen's *d*, Pearson's *r*), indicating how they were calculated

Our web collection on statistics for biologists contains articles on many of the points above.

Software and code

Policy information about availability of computer code

Data collection	All the MS data were collected using Thermo Scientific Xcalibur software (version 4.1.50). All fluorescence images were collected using Olympus Cell Sens Standard (Version 1.12). Western blot images were collected using a VersaDoc imaging station (Bio-Rad) (version 2.4.0.03) or Epson Scan (Version 3.9.2.4us) with an Epson Perfection scanner (V37).
Data analysis	pFind (Version 3.0.11, http://pfind.ict.ac.cn/software/pFind3/index.html) was used for analyzing MS data.<br>Origin (Version 8, http://www.OriginLab.com) was used for making scatter plots.<br>Excel 2016 (https://products.office.com/en/excel) was used for making pie charts, bar charts.<br>BoxPlotR (http://shiny.chemgrid.org/boxplotr, there is no version # for this tool) was used for making a violin plot.<br>DAVID (version 6.8, https://david.ncifcrf.gov/home.jsp) was used for GO and KEGG analyses.<br>Image J (version Java 1.6.0_24 (64-bit)) was used to analyze Western blots and fluorescent images.<br>GraphPad Prism (Version 8.4.2) was used for statistics.

For manuscripts utilizing custom algorithms or software that are central to the research but not yet described in published literature, software must be made available to editors and reviewers. We strongly encourage code deposition in a community repository (e.g. GitHub). See the Nature Research guidelines for submitting code & software for further information.

Data

Policy information about availability of data

All manuscripts must include a data availability statement. This statement should provide the following information, where applicable:

- Accession codes, unique identifiers, or web links for publicly available datasets
- A list of figures that have associated raw data
- A description of any restrictions on data availability

The mass spectrometry proteomics data have been deposited to the ProteomeXchange Consortium via the PRIDE partner repository (Project accession: PXD018575, http://www.ebi.ac.uk/pride/archive/projects/PXD018575).
C. elegans Uniprot canonical database can be downloaded using the following link (https://www.uniprot.org/proteomes/UP000001940)

Field-specific reporting

Please select the one below that is the best fit for your research. If you are not sure, read the appropriate sections before making your selection.

[x] Life sciences [ ] Behavioural & social sciences [ ] Ecological, evolutionary & environmental sciences

For a reference copy of the document with all sections, see nature.com/documents/nr-reporting-summary-flat.pdf

Life sciences study design

All studies must disclose on these points even when the disclosure is negative.

Sample size	2-3 independent samples were prepared for all MS-based proteomic experiments to meet the established standards of the field of redox proteomics (Martell J et al. Cel Chem Biol, 2016; Alkter S et al. Nat Chem Biol. 2018; Petrova B, et al., PNAS, 2018; Huang J et al., PNAS, 2019). 2-3 independent samples were prepared for all WB experiments and qRT-PCR experiments, which is common (Chamoli et al., Nat Commun, 2020; Topf et al., Nat Commun, 2018; Wu et al., Cell Metabolism, 2019; Su et al., Nat Commun, 2019).<br>For fluorescence microscopy, 3 independent experiments containing a total of at least 100 worms were conducted.<br>For Pathogenesis Assays, 2 independent experiments containing a total of 280-388 worms per condition were analyzed, which is common in this field (Wu et al., Cell Metabolism, 2019; Fletcher et al., Plos Genetics, 2019).
Data exclusions	No data were excluded from the analyses.
Replication	Both proteomic and functional experiments were performed in duplicates or triplicates. Number of repeats performed for each experiments are included in figure legends. Data can be reproduced if necessary.
Randomization	Not applicable, because there were no other variations and experimental conditions were tightly controlled.
Blinding	For mass spectrometry analyses, data collection were single-blinded. For functional assays, investigators were not blinded. Blinding is not typically done for survival analyses, Western blots, and qRT-PCR. Western blot images and qRT-PCR results were obtained and analyzed by commercial software. For survival analyses and fluorescence microscopy, investigators were careful to maintain objectivity and used clearly defined scoring criteria (mentioned in Methods).

Behavioural & social sciences study design

All studies must disclose on these points even when the disclosure is negative.

Study description	*Briefly describe the study type including whether data are quantitative, qualitative, or mixed-methods (e.g. qualitative cross-sectional, quantitative experimental, mixed-methods case study).*
Research sample	*State the research sample (e.g. Harvard university undergraduates, villagers in rural India) and provide relevant demographic information (e.g. age, sex) and indicate whether the sample is representative. Provide a rationale for the study sample chosen. For studies involving existing datasets, please describe the dataset and source.*
Sampling strategy	*Describe the sampling procedure (e.g. random, snowball, stratified, convenience). Describe the statistical methods that were used to predetermine sample size OR if no sample-size calculation was performed, describe how sample sizes were chosen and provide a rationale for why these sample sizes are sufficient. For qualitative data, please indicate whether data saturation was considered, and what criteria were used to decide that no further sampling was needed.*
Data collection	*Provide details about the data collection procedure, including the instruments or devices used to record the data (e.g. pen and paper, computer, eye tracker, video or audio equipment) whether anyone was present besides the participant(s) and the researcher, and whether the researcher was blind to experimental condition and/or the study hypothesis during data collection.*
Timing	*Indicate the start and stop dates of data collection. If there is a gap between collection periods, state the dates for each sample cohort.*

Data exclusions	*If no data were excluded from the analyses, state so OR if data were excluded, provide the exact number of exclusions and the rationale behind them, indicating whether exclusion criteria were pre-established.*
Non-participation	*State how many participants dropped out/declined participation and the reason(s) given OR provide response rate OR state that no participants dropped out/declined participation.*
Randomization	*If participants were not allocated into experimental groups, state so OR describe how participants were allocated to groups, and if allocation was not random, describe how covariates were controlled.*

Ecological, evolutionary & environmental sciences study design

All studies must disclose on these points even when the disclosure is negative.

Study description	*Briefly describe the study. For quantitative data include treatment factors and interactions, design structure (e.g. factorial, nested, hierarchical), nature and number of experimental units and replicates.*
Research sample	*Describe the research sample (e.g. a group of tagged Passer domesticus, all Stenocereus thurberi within Organ Pipe Cactus National Monument), and provide a rationale for the sample choice. When relevant, describe the organism taxa, source, sex, age range and any manipulations. State what population the sample is meant to represent when applicable. For studies involving existing datasets, describe the data and its source.*
Sampling strategy	*Note the sampling procedure. Describe the statistical methods that were used to predetermine sample size OR if no sample-size calculation was performed, describe how sample sizes were chosen and provide a rationale for why these sample sizes are sufficient.*
Data collection	*Describe the data collection procedure, including who recorded the data and how.*
Timing and spatial scale	*Indicate the start and stop dates of data collection, noting the frequency and periodicity of sampling and providing a rationale for these choices. If there is a gap between collection periods, state the dates for each sample cohort. Specify the spatial scale from which the data are taken*
Data exclusions	*If no data were excluded from the analyses, state so OR if data were excluded, describe the exclusions and the rationale behind them, indicating whether exclusion criteria were pre-established.*
Reproducibility	*Describe the measures taken to verify the reproducibility of experimental findings. For each experiment, note whether any attempts to repeat the experiment failed OR state that all attempts to repeat the experiment were successful.*
Randomization	*Describe how samples/organisms/participants were allocated into groups. If allocation was not random, describe how covariates were controlled. If this is not relevant to your study, explain why.*
Blinding	*Describe the extent of blinding used during data acquisition and analysis. If blinding was not possible, describe why OR explain why blinding was not relevant to your study.*

Did the study involve field work? ☐ Yes ☐ No

Field work, collection and transport

Field conditions	*Describe the study conditions for field work, providing relevant parameters (e.g. temperature, rainfall).*
Location	*State the location of the sampling or experiment, providing relevant parameters (e.g. latitude and longitude, elevation, water depth).*
Access & import/export	*Describe the efforts you have made to access habitats and to collect and import/export your samples in a responsible manner and in compliance with local, national and international laws, noting any permits that were obtained (give the name of the issuing authority, the date of issue, and any identifying information).*
Disturbance	*Describe any disturbance caused by the study and how it was minimized.*

Reporting for specific materials, systems and methods

We require information from authors about some types of materials, experimental systems and methods used in many studies. Here, indicate whether each material, system or method listed is relevant to your study. If you are not sure if a list item applies to your research, read the appropriate section before selecting a response.

Materials & experimental systems

n/a	Involved in the study
☐	☒ Antibodies
☒	☐ Eukaryotic cell lines
☒	☐ Palaeontology and archaeology
☐	☒ Animals and other organisms
☒	☐ Human research participants
☒	☐ Clinical data
☒	☐ Dual use research of concern

Methods

n/a	Involved in the study
☒	☐ ChIP-seq
☒	☐ Flow cytometry
☒	☐ MRI-based neuroimaging

Antibodies

Antibodies used

Anti-actin antibody (cat #: A5441) and anti-tubulin antibody (cat #: T9026) were purchased from Sigma-Aldrich; Anti-p-eIF2α (cat #: 3597) and anti-p-p38 (cat #: 9211) antibodies were purchased from Cell Signaling Technology; Anti-PMK-1 antibody was made by Dr. R. Pukkila-Worley at University of Massachusetts Medical School, Worcester, MA (Thermo Scientific Pierce Custom Antibody Services, according to Peterson et al., Plos Genetics, 2019). Anti-PRDX-2 antibody was made by Dr. E. Veal at Newcastle University Biosciences Institute, Newcastle upon Tyne, UK (Eurogentec, according to Ola´ hova´ et al., PNAS, 2008).

Validation

The commercial antibodies used in this study include anti-actin, anti-tubulin, anti-p-eIF2α and anti-p-p38 antibodies. All these antibodies have been validated in multiple species. These antibodies recognize evolutionarily conserved sites, and have been routinely used in C. elegans. Publications that have used these antibodies include: anti-actin: Wu et al., Cell Metabolism, 2019; anti-tubulin and anti-p-p38: Hourihan et al., Molecular Cell, 2016; anti-p-eIF2α: Baker et al., Plos Genetics, 2012.

Animals and other organisms

Policy information about studies involving animals; ARRIVE guidelines recommended for reporting animal research

Laboratory animals

The C. elegans strains Wild-type (N2), TJ356: zIs356(daf-16p::daf-16a/b::GFP;rol-6(su1006)), pmk-1(km25), sek-1(km4) were obtained from Caenorhabditis Genetics Center. LD1758 was obtained by backcrossing pmk-1(km25) 5X to N2, whereas LD1757 was obtained by backcrossing sek-1(km4) 4X to N2. Alleles pmk-1(syb1415) and sek-1(syb1398) were generated by SunyBiotech (China). LD1906 was obtained by backcrossing pmk-1(syb1415) 4X to N2, and LD1886 was obtained by backcrossing sek-1(syb1398) 4X to N2. Experiments were conducted using L4 stage worms unless specified. All animals used were hermaphrodites.

Wild animals

No wild animals were used in this study.

Field-collected samples

No field-collected samples were used in this study.

Ethics oversight

This study did not require an ethical approval.

Note that full information on the approval of the study protocol must also be provided in the manuscript.
